# FBH1: A new checkpoint factor

**DOI:** 10.18632/oncotarget.5099

**Published:** 2015-08-01

**Authors:** Anne-Sophie Boyer, Claus Storgaard Sørensen

**Affiliations:** Biotech Research and Innovation Centre (BRIC), University of Copenhagen, Copenhagen, Denmark

Unraveling the machineries that deal with threats during DNA replication is of critical importance to understand how cells maintain genome stability. Faithful duplication of the genome is fundamental for the propagation of life, yet DNA is particularly vulnerable during the replication process. DNA replication forks encounter a large number of obstacles in the template DNA in each cell cycle. These perturbations lead to fork stalling and if not quickly resolved the fork can collapse through disassembly of the replication machinery. In case of widespread stalling of forks cells enter a state commonly referred to as ‘replicative stress’ that challenge genome stability. Cellular mechanisms have evolved to monitor this process and ensure completion of genome replication by different means. Following replication stress, cell cycle checkpoint pathways are activated to stabilize stalled replication forks and halt cell cycle progression. Genome duplication is then completed by employing several pathways, which include the activation of nearby dormant replication origins, the repriming of DNA replication downstream of the damage site, and tolerance to the damage thanks to lesion bypass mediated by specialized DNA polymerases [1].

The mechanisms underlying critical processes at the DNA replication fork still remain to be fully elucidated, and new important factors are emerging. The FBH1 helicase is a less investigated regulator of the response to stalled replication forks. FBH1 is a member of the conserved UvrD family of 3′-5′ DNA helicases [2] that has been shown to operate on a number of cellular substrates including stalled replication forks. FBH1 accumulates at stalled forks shortly after exposure to hydroxurea (HU), a chemotherapeutic agent inducing replication stress by depleting the pool of dNTPs required for DNA synthesis. In our laboratory, we have recently identified FBH1 as the first enzyme responsible for fork regression *in vivo* in higher eukaryotes [3], an activity observed following hydroxyurea-induced stalling of forks. Replication fork regression is the process where a replication fork is converted to a four-way DNA junction. This is achieved by coordinated annealing of the two newly synthesized strands at the fork, the leading and the lagging strands of DNA replication. This new annealed DNA double-strand form the fourth arm of the four-way DNA junction [4]. Importantly, FBH1 also possesses fork regression activity *in vitro* supporting that FBH1 is a *bona fide* fork regression enzyme in mammalian cells [3].

What is the role of FBH1-mediated replication fork regression? This process can either be an end product or intermediate in fork processing reactions. As an end product, regression may stabilize the fork for example by shielding vulnerable single-stranded DNA regions. This can provide additional genomic safety and time for neighboring forks to complete DNA replication. The data at hand also suggest that regressed forks may elicit signals to signify that severe replication stress is present. Notably, the very end of the regressed arm of the four-way junction is a DNA double-stranded end, which has obvious similarity to the DNA double-strand break (DSB) generated for example after ionizing radition. In line with this notion, whereas replication fork stalling does not induce detectable DNA DSB formation immediately after HU treatment, we observed that HU nonetheless induces a marked FBH1-dependent DNA DSB-like signaling response, which is already evident 1-2 hours following HU treatment. This response can be measured by the marked phosphorylation of DNA DSB-related ATM targets before detectable DSBs were apparent [3]. Thus, our data imply a conceptually new signaling pathway, whereby stalled forks are processed by FBH1 to form DNA structures with the ability to signal like DNA DSB end structures. Intiguingly, Fork regression *per se* may be insufficient to activate the checkpoint, as fork regression and replication stress do not always co-occur [4,5]. It is possible that DNA DSB-independent ATM signaling upon fork stalling is dependent on specific molecular features of the induced reversed forks, where fork stalling due to nucleotide shortage may differ from fork stalling due to template lesions. Hence, ATM signaling from double stranded ends at regressed arms might require binding of cellular mediators or removal of replicative factors, which may only occur upon specific conditions of replication stress.

Strikingly, the response to long-term fork stalling also requires FBH1. When replication forks are stalled more than 12-16 hours with HU, there is measurable cleavage of the fork catalyzed by the structure-specific endonuclease MUS81, which requires FBH1 helicase activity. This reaction generates double-stranded DNA ends that can be used for recombination-dependent replication fork restart. However, the MUS81-induced DNA breakage triggers p53 activation that ultimately also serves to suppress cell proliferation untill replication stress has been eliminated [6, 7].

Based on our understanding so far, FBH1-mediated DNA replication fork processing is emerging as a new pathway for genome protection in response to replication stress. Future studies will aim to unravel how this pathway interacts with other fork processing and signaling pathways.
